# Elite Model for the Generation of Induced Pluripotent Cancer Cells (iPCs)

**DOI:** 10.1371/journal.pone.0056702

**Published:** 2013-02-13

**Authors:** Jason Lai, Chiou Mee Kong, Dashayini Mahalingam, Xiaojin Xie, Xueying Wang

**Affiliations:** Department of Biochemistry, Yong Loo Lin School of Medicine, National University of Singapore, Singapore, Singapore; University of Kansas Medical Center, United States of America

## Abstract

The inefficiency of generating induced pluripotent somatic cells (iPS) engendered two contending models, namely the Stochastic model and Elite model. Although the former is more favorable to explain the inherent inefficiencies, it may be fallible to extrapolate the same working model to reprogramming of cancer cells. Indeed, tumor cells are known to be inherently heterogeneous with respect to distinctive characteristics thus providing a suitable platform to test whether the reprogramming process of cancer cells is biased. Here, we report our observations that all randomly picked induced pluripotent cancer cells (iPCs) established previously do not possess mutations known in the parental population. This unanticipated observation is most parsimoniously explained by the Elite model, whereby putative early tumor progenies were selected during induction to pluripotency.

## Introduction

### Induced Pluripotent Cancer Cells (iPCs)

Induction of cancer cells to pluripotency (iPCs) has been successfully achieved on various cancer cells and showed promising results of attenuating their tumorigenicity [1]–[5]. However, given that each established pluripotent cancer cell colony is assumed to be clonal from a single parental cancer cell, and that the parental cancer cell population is likely heterogeneous, it will be of poignant interest to understand whether the nuclear reprogramming process is biased. Although Yamanaka (2009) proposed that the generation of induced pluripotent stem cells (iPS) is not a biased process (Stochastic model) [6], it is indeed fallible to extrapolate this model to the generation of iPCs, given the heterogeneity of cancer cells.

### Intratumor heterogeneity

Individual tumors have commonly been observed to be morphologically and karyotypically heterogeneous [7]–[12], occasionally obscuring histopathologists in accurately determining the tumor grade for clinical diagnosis. Furthermore, a classic subcloning experiment conducted by Fidler, I.J. and Kripke, M.L. provided compelling evidence that heterogeneity within a tumor exists with respect to metastatic ability [13]. Moreover, the aggressive advancement of next-generation sequencing (NGS) has already ushered in the possibility of single nucleus sequencing, which proposed a punctuated model for tumor evolution [14].

To test whether the Stochastic model holds in reprogramming of cells, a distinguishable heterogeneous population should be utilized to observe whether any subpopulation is over-represented in reprogrammed colonies. Indeed, this condition is fulfilled by cancer cells, which presents an interesting experimental setup. Hochedlinger *et al.* (2004) observed that reprogrammed mice melanoma cells share a homogenous karyotype configuration, in contrast to its parental cell population that is heterogeneous [15]. Indeed, if the Stochastic model holds, the karyotype of the reprogrammed melanoma population should be heterogeneous between clones. However, a critical parameter to fully substantiate the rejection of the Stochastic model needs to be determined: the proportion of parental cells that possess the same karyotype configuration as the reprogrammed cells. If this proportion is large in the prior, statistically, there is little basis to reject the Stochastic model in this instance. However, if the proportion is sufficiently small, it is indeed perceivable to reject the Stochastic model.

Here, we report similar observations on the reprogramming of two non-small cell lung cancer (NSCLC) cells – H358 and H460. The former was reported to be *TP53* homozygous deleted [16], the latter carries homozygous deleted *CDKN2A* [17] and mutant *CDKN2B* (unpublished data). Quite surprisingly, we observed that iPCs generated from these cancer cells, i.e., iPCH358 and iPCH460, no longer harbor any of the known deletions or mutations. Moreover, *TP53* observed in iPCH358 and *CDKN2A, CDKN2B* in iPCH460 were observed to be wild-type. Furthermore, the aforementioned critical parameter was determined and suggests the rejection of the Stochastic model; our experimental results suggest that reprogramming of cancer cells follows the Elite model which has selected a distinct subpopulation of cells from a heterogeneous parental cancer cell population.

## Materials and Methods

### Cell lines and culture

Cell lines used in this study are human fetal lung fibroblast IMR90 (ATCC no. CCL-186), HeLa (ATCC no. CCL-2), adenocarcinoma NCI-H358 (ATCC no. CRL-5807), large cell carcinoma NCI-H460 (ATCC no. HTB-177), as well as human embryonic stem cell H1 (WiCell no. WA01). NCI-H460 obtained from Dr. Koeffler's laboratory was also purchased from ATCC. All cell lines were maintained in humidified incubator maintained at 5% CO_2_ and 37°C cultured in ATCC recommended media supplemented with 10% FBS. The generation of iPCs was described previously [18]. Briefly, iPCs were established via Yamanaka's protocol with slight modification [19]. Lentivirus and retrovirus were produced by transfecting 293T cells and Plat-E cells, respectively. Prior to infecting H358 and H460 cells, viruses were filtered through a 0.45 µm pore-size surfactant-free cellulose acetate filter (Sartorius). iPCs and hESC were cultured on irradiated mouse embryonic fibroblast (iMEFs) bathed in DMEM/F12 (Invitrogen) supplemented with 20% Knockout Serum Replacement (Invitrogen), 1 mM L-glutamine (Invitrogen), 100 µM nonessential amino acids, 100 µM β-mercaptoethanol (Sigma-Aldrich) and 4 ng/µL basic fibroblast growth factor (bFGF) (Invitrogen). Prior to conducting experiments on iPCs and hESC, cells were seeded on Matrigel (BD Bioscience) and maintained in mTeS®1 (STEMCELL Technologies).

### RNA/DNA isolation and reverse transcription PCR (RT-PCR)

Total RNA was extracted using RNeasy Mini Kit (Qiagen) and reverse-transcribed using Reverse Transcriptase Enzyme (Promega) as well as oligo dT primers (Promega), according to manufacturer's instruction. Total DNA was extracted using DNeasy Blood & Tissue Kit (Qiagen).

### Microarray Analysis

Microarray data for gene expression and DNA methylation were obtained from GSE35913. The analyses were performed using R in lumi and methylumi environments [20]. Heat map was generated using gplots package.

### PCR and sequencing

*TP53*, *CDKN2A* and *CDKN2B* were amplified from cDNA and genomic DNA of IMR90 (positive control), H358, H460 and 10 randomly picked iPCH358 and iPCH460 colonies. Primers for amplifications can be found in Table S1. PCR products were resolved by gel electrophoresis and purified with QIAquick Gel Extraction Kit (Qiagen) and sequenced using BigDye® Terminator v3.1 cycle sequencing kit (Applied Biosystems). The PCR product for *TP53* amplicon 2 for iPCH358 Col#3 was cloned onto pGEM®-T vector (Promega) before sequencing.

### Western Blot

Whole cell lysates of H1, HeLa, H358, H460 and 10 randomly picked iPCH358 and iPCH460 colonies each were resolved by SDS-PAGE and transferred to a nitrocellulose membrane. TP53 primary antibody (Cell Signaling #9282) was used to probe presence of TP53 in H1, HeLa, H358 and all iPCH358 colonies. CDKN2A primary antibody (Cell Signaling #4824) was used to probe presence of CDKN2A in H1, HeLa, H460 and all iPCH460 colonies.

### Serial Dilution Assay

30 ng/µL of IMR90 genomic DNA was serially diluted 10-fold by 30 ng/µL of H460 genomic DNA. 1 µL of the concoction was then used as template for the amplification of *CDKN2B*.

### Explant tumors

About 2×10^6^ H358 and H460 cells were resuspended in 30% Matrigel and injected subcutaneously into severe combined immunodeficient (SCID) mice or nude mice (Table S2). Tumors were allowed to grow for three to four weeks. Mice were sacrificed before excision of tumors. The animal experiments were performed under approved IACUC (The National University of Singapore Institutional Animal Care and Use Committee) protocols of 117/09.

### Metaphase spread

Metaphase spreads of parental and post-iPC was performed as described by Jeppesen [21]. Briefly, cells were cultured to 70% confluency and were treated with 0.1 µg/ml of Demecolcine solution (Sigma) for 7 hours. Cells were then dissociated with trypsin and treated with 75 mM of KCl at 37°C for 10 minutes. 5×10^3^ cells (in 100–500 µl KCl) were spun at 1000 rpm to the cytocentrifuge slides for 5 minutes. Slides were then washed with KCM (120 mM KCl, 20 mM NaCl, 10 mM Tris-HCl pH7.5, 0.5 mM EDTA, 0.1% Triton X-100) for 5 minutes, blocked with 10% BSA (diluted in KCM) for 45 minutes. This is followed by incubation with primary antibodies for two hours and then fluorescence-conjugated secondary antibodies for one hour. After washing the slides with KCM, the spreads were fixed with 4% formaldehyde for 15 minutes. Finally, slides were washed with distilled water, air-dried and mounted with mounting medium containing DAPI (Vector Laboratories). All images were captured using Olympus Fluoview FV1000 microscope. Primary antibodies used were *CENPA* (Abcam) and *TRF2* (BD Transduction Laboratories).

### Accession numbers

All sequencing data of *TP53*, *CDKN2A* and *CDKN2B* were uploaded to GenBank under accession numbers JQ694043-51and JX391994.

## Results

### Presence of *TP53* observed in iPCH358

**Figure 1:**
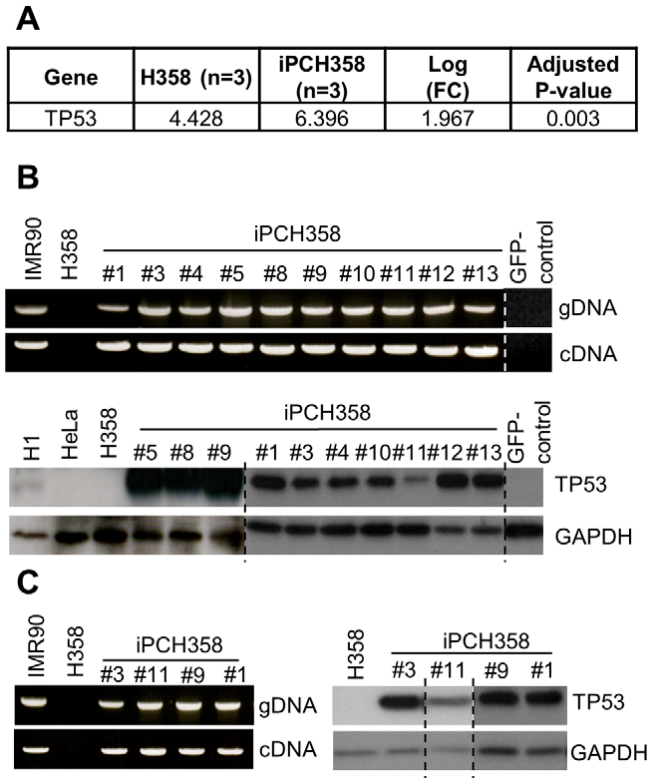
Unexpected expression of wild-type *TP53* in iPCH358 but not in H358. (A) log-transformed intensity readouts from Illumina HumanHT-12 indicating a significant (FDR-adjusted P<0.05) upregulation of *TP53* transcript in iPCH358 compared to H358. This is unexpected since H358 is known to be *TP53*−/−. (B) PCR (upper panel) and Western Blot (lower panel) assays confirming expression of *TP53* in several randomly picked colonies of iPCH358. The coding region of *TP53* in Col#1, Col#3 and Col#11 are wild-type (GenBank accession: JQ694049–JQ694051). (C) PCR (left panel) and Western Blot (right panel) assays on iPCH358 colonies of >20 passages revealed that passage number is uninformative to the outcome of these assays. Results from experiments conducted on separate occasions or cropped from the same image are marked by a broken line; original images can be found in Presentation S1 which includes detailed documentation of passage number.

The successful establishment of iPCH358 and iPCH460 in our laboratory was reported previously [18]. Gene expression microarray data revealed that detection of *TP53* expression in H358 was similar to background noise levels for all three biological replicates, but upregulated in iPCH358, which was entirely unexpected (Figure 1A). To rule out microarray artifact, *TP53* in 10 randomly picked iPCH358 colonies was interrogated by PCR and Western Blot. To our surprise, these assays unanimously agree with the microarray data (Figure 1B). Furthermore, we sequenced the coding region of *TP53* and found it to be wild-type in three randomly picked colonies of iPCH358 (GenBank accession: JQ694049–JQ694051).

### *CDKN2A* and *CDKN2B* not mutated in iPCH460

**Figure 2:**
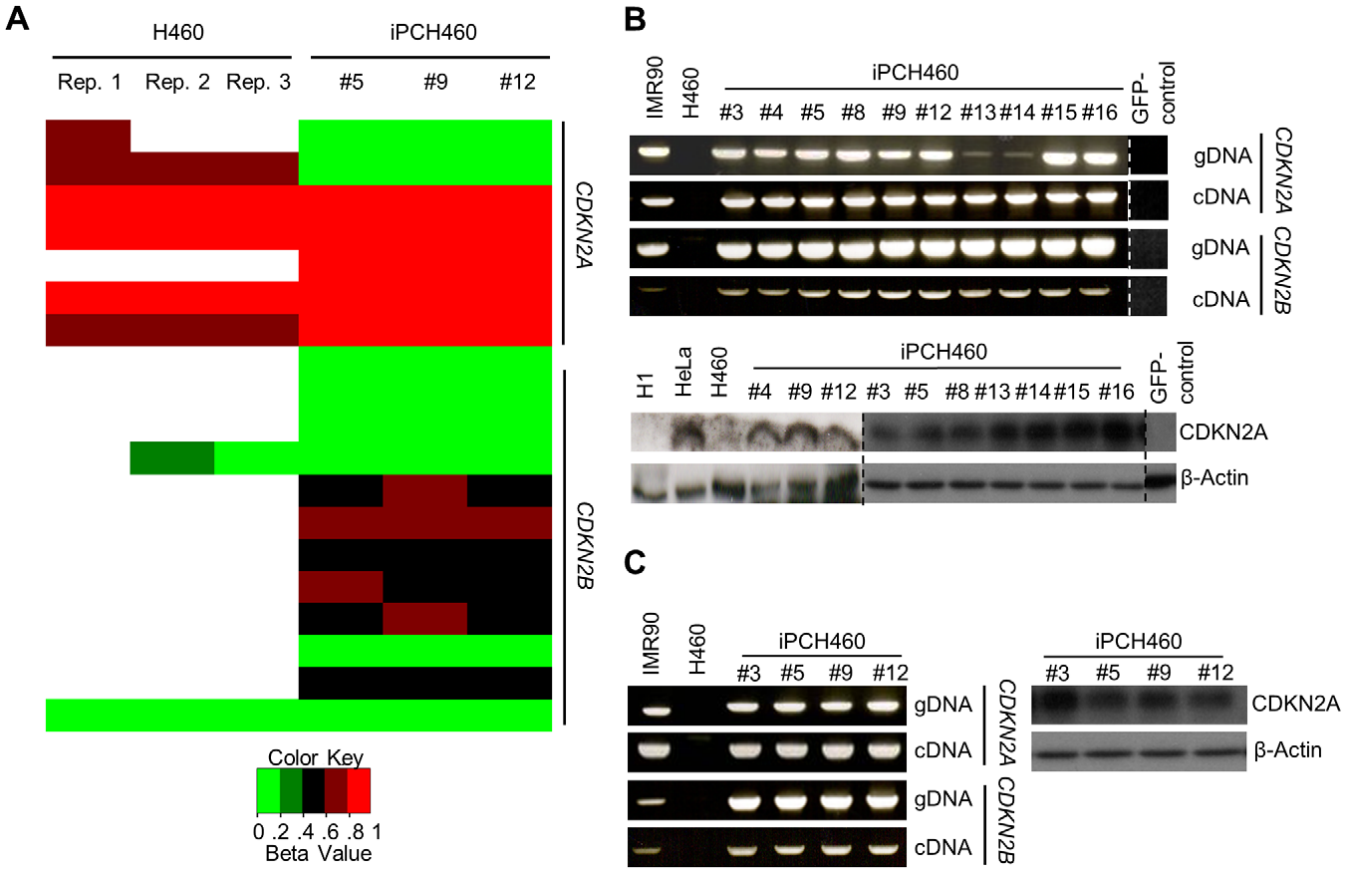
*CDKN2A* and *CDKN2B* are not mutated in iPCH460 colonies. (A) Heat map indicating methylation array probes (rows) failing to hybridize to *CDKN2A* and *CDKN2B* promoters in H460 (empty bars), but not so in iPCH460. (B) PCR (upper panel) assay showing *CDKN2A* and *CDKN2B* are detectable in iPCH460 while Western Blot (lower panel) assay showing *CDKN2A*, which is homozygous deleted in H460, is detectable in iPCH460. (C) PCR (upper panel) and Western Blot (lower panel) assays on iPCH460 colonies of >20 passages revealed that passage number is uninformative to the outcome of these assays. Results from experiments conducted on separate occasions or cropped from the same image are marked by a broken line; original images can be found in Presentation S1 which includes detailed documentation of passage number.

This result motivated us to determine whether a similar occurrence was observed in H460. Probe sets (DNA methylation microarray) for the promoter of *CDKN2A* (*P16*) and *CDKN2B* (*P15*) were not able to produce reliable signals for all biological replicates (n = 3) of H460, which is likely due to mutations at the interrogated loci. However, the same cannot be said for iPCH460 (Figure 2A). To validate this observation, primer pairs designed by Shan, *et al.* (2004), which will not yield any PCR products from H460 genomic DNA template, were utilized [22]. Surprisingly, the same primer pair was able to produce PCR products from 10 randomly picked colonies of iPCH460 genomic DNA template (Figure 2B). Similarly, the primer pairs designed to amplify the coding region of both *CDKN2A* and *CDKN2B* mRNA did not yield any PCR products from H460, but all 10 iPCH460 colonies yielded PCR products under the same conditions (Figure 2B). Moreover, sequencing results of the coding regions of both *CDKN2A* and *CDKN2B* mRNA of three randomly picked iPCH460 colonies were observed to be wild-type (GenBank accession: JQ694043–JQ694048). In addition, *CDKN2A* that is known to be deleted in H460 is expressed and detectable by Western Blot in iPCH460 (Figure 2B). Although no PCR products from amplifying *CDKN2B* in H460 were observed, CDKN2B protein is actively expressed in H460 and remained so in iPCH460 (Figure S1). Our laboratory discovered that instead of whole gene deletion, putative small mutations in the surrounding loci of *CDKN2B* in H460 rendered established PCR methods to fail. This was verified with independently sourced H460 cells from Dr. Koeffler's laboratory (Figure S2).

### Expression of deleted genes persists through late passages of iPC colonies

Chin and colleagues reported that early passages (≤10 passages) of iPS colonies behaved differently from their late passages (>20 passages) counterparts [23]. In our earlier assays (Figure 1B and Figure 2B), our samples were predominantly ≤20 passages. Therefore, we proceeded to characterize if passage number will modify the expression behavior of *TP53* in iPCH358 as well as *CDKN2A* and *CDKN2B* in iPCH460. Interestingly, we did not observe any change in expression behavior in late passage iPCs (Figure 1C, Figure 2C and Presentation S1).

A possible confounder to our observation thus far is the presence of contaminating normal fibroblasts in these cancer cell lines. Therefore, to rule out the possibility that these resultant iPCs were derived from contaminating normal fibroblasts, metaphase spread was conducted on post-iPC cells (spontaneously differentiated iPC colonies via embryoid body formation). We observed that the post-iPC cells remain aneuploid and thus conclude that the established iPC colonies were not derived from contaminating normal fibroblasts (Figure S3). We also ruled out 293T cell and Plat-E cell contamination because both lentivirus and retrovirus were filtered through a 0.45 µm pore-size filter before infecting H358 and H460. In addition, we found that the GFP-control vectors played no role in the expression of the mutated genes in both H358 and H460 (Figure 1B and Figure 2B).

Taken together, we have here two complementing hypotheses to explain why *TP53* null H358 cells expressed TP53 upon reprogramming (likewise, *CDKN2A* null H460 cells expressed CDKN2A upon reprogramming): 1) reprogramming induces a gene recovery mechanism; 2) reprogramming, in a heterogeneous population, enriched a subpopulation of cells with different mutational status than the majority population. Indeed, the latter hypothesis provides the most parsimonious explanation (see discussion).

### Elite model of reprogramming predicts our observations

**Figure 3:**
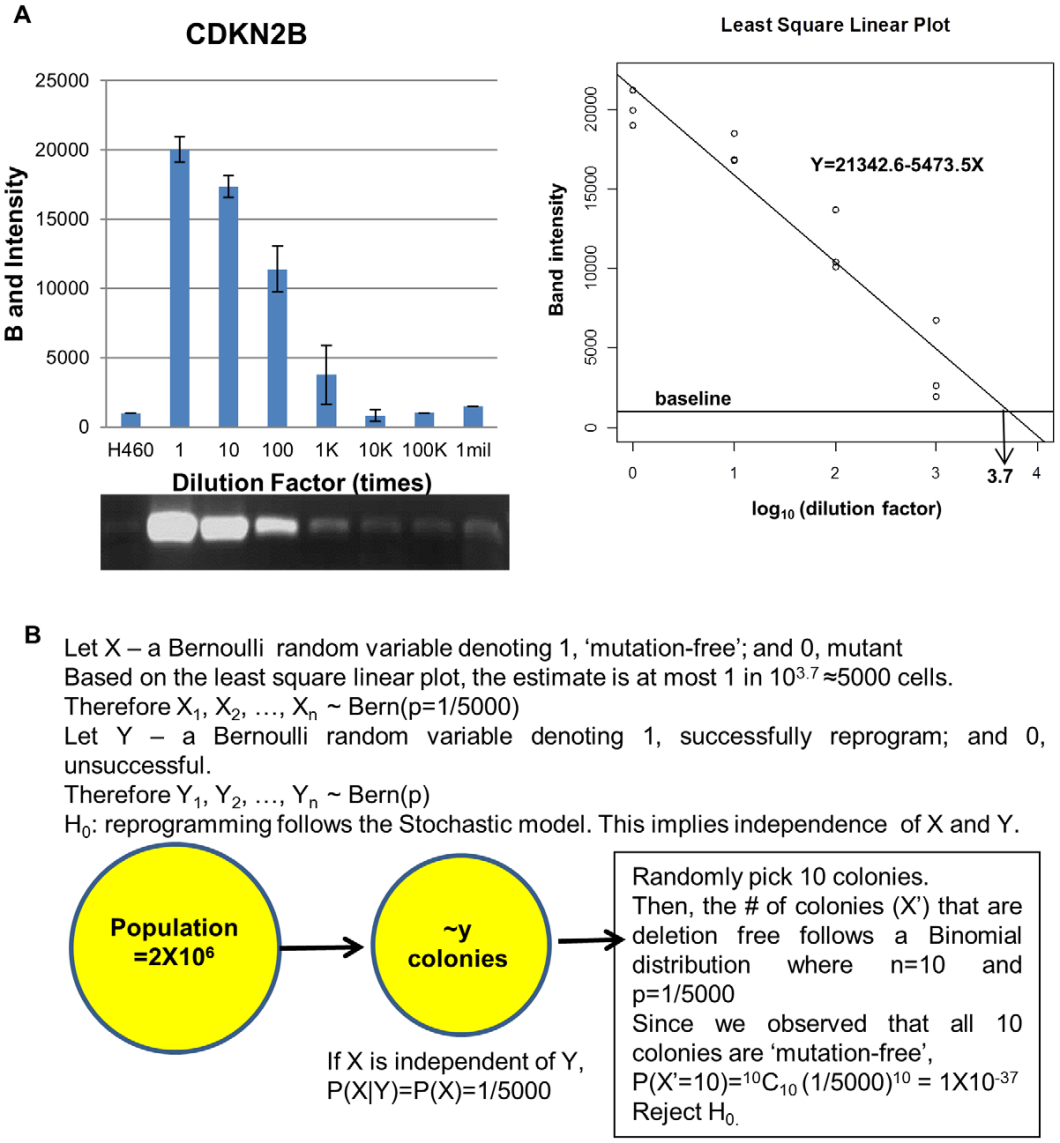
Evidence rejecting the Stochastic model for generating iPCs. (A) Serial dilution assay showing that at 10^3.7^ times dilution, *CDKN2B* in IMR90 is detectable at basal levels. Therefore, at most 1 in 5000 H460 cells are ‘mutation-free’. (B) The probability model to test the null hypothesis: “Generation of iPCs follows the Stochastic model”. Subsequent input of the parameters determined in (A) suggests the rejection of the Stochastic model in the reprogramming of H358 and H460.

Given that H358 and H460 are heterogeneous with respect to gene mutation status, we asked, “What is the probability that all randomly picked iPCs colonies are derived from the minor subpopulation without known mutation that characterizes the majority population, if reprogramming follows the Stochastic model?” We first established one parameter: The estimated proportion of the minor subpopulation (for simplicity, this subpopulation will be referred to as the ‘mutation-free’ subpopulation). To estimate it, IMR90 genome was serially diluted with H460 genome and assessed the efficacy to amplify *CDKN2B* by PCR. We chose to amplify *CDKN2B* due to its amplification efficiency compared to *CDKN2A* or *TP53* (Figure S4). In our assay, we estimated that the maximum proportion of H460 cells without mutant *CDKN2B* is 1∶5000 (Figure 3A). With this estimate, we calculated the probability of observing all randomly picked colonies that were derived from the ‘mutation-free’ subpopulation. For both iPCH358 and iPCH460, the probability of observing all 10 randomly picked colonies to be ‘mutation-free’ is 1×10^−37^ (Figure 3B). In addition, the smallest possible proportion to result in a probability of 0.05 or more in this probability model is 0.75 (^10^C_10_×0.75^10^×0.25^0^>0.05), i.e. if the starting population had no less than three ‘mutation-free’ cells for every four cells. This proportion is not anywhere near 0.25, which is the poorest estimated proportion from the least efficient amplification of the serial dilution assays (Figure S4A). Thus, we reject the null hypothesis and conclude that reprogramming of cancer cells follows the Elite model.

### Defining the ‘mutation-free’ subpopulation

**Figure 4:**
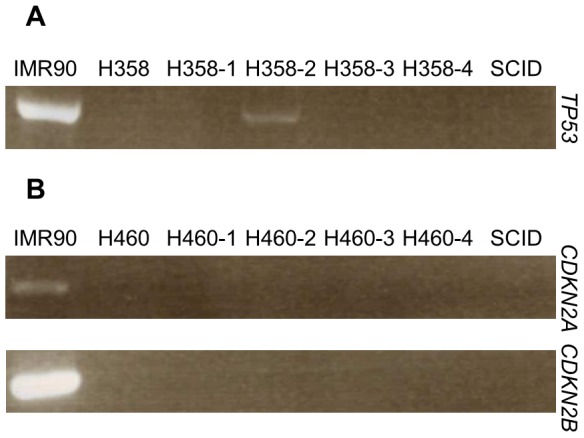
Single explant tumor of H358 enriches ‘mutation-free’ subpopulation. (A) One out of four H358 explant tumor generated show presence of *TP53* in the genome, indicating enrichment of the elusive ‘mutation-free’ subpopulation. (B) None of the H460 explant tumors were observed to be enriched for ‘mutation-free’ subpopulation. Genomic DNA of SCID mice tail was used to control for mice DNA contamination in explant tumors. Information of explant tumors can be found in Table S2.

While the Elite model in this reprogramming experiment asserts that the process is biased towards the ‘mutation-free’ subpopulation, the enrichment of this elusive subpopulation in its native state is technically challenging. Nonetheless, Hochedlinger *et al.* (2004) previously showed that the karyotype configuration of the reprogrammed cancer cell is similar to that of the explant tumor [15], suggesting that inoculation of cancer cell line in SCID mice has selected a tumorigenic subpopulation with cytogenetic feature similar to that of the reprogrammed cancer cell. In addition, it was pointed out that Chang *et al.* (2003) observed that tumor cell lines were typically heterogenous in contrast to their derivative explant tumors [24]. Hence, four explant tumors of H358 and H460 were generated each (Table S2). However, only one explant tumor of H358 (H358-2) yielded detectable *TP53* on its genomic DNA (Figure 4A). On the other hand, none of the explant tumors of H460 yielded detectable *CDKN2A* or *CDKN2B* (Figure 4B). Therefore, inoculation of cancer cell line in SCID mice weakly emulates the selectivity of nuclear reprogramming.

## Discussion

### Reprogramming discriminates heterogeneous cancer cell population

The data presented here show that the genetic mutation status differs between the parental cancer cell population and the reprogrammed counterpart. While we do not have experimental data to show the homogeneity or heterogeneity of H358 and H460 cancer cell populations directly, in order to explain this intriguing observation, we proposed two complementing hypotheses: 1) The starting population is homogenous and thus nuclear reprogramming inadvertently corrects certain mutations; 2) The starting population is heterogeneous and nuclear reprogramming enriches a minor subpopulation. The first hypothesis hinges on an assumption whereby a cell is capable of recovering a mutated gene. However, it is clear that such a mechanism does not exist because establishment of iPS from *p53, Terc* and *Ink4/Arf* knock-out mouse embryonic fibroblasts [25], [26], did not see the recovery of these genes. On the other hand, the second hypothesis assumes a scenario whereby reprogramming discriminates within the heterogeneous cancer cell population of varying degrees of genetic insult. This assumption closely resembles to the observation Hochedlinger and colleagues made whereby the heterogeneous karyotype of the mice melanoma becomes homogenous post-reprogramming [15]. Therefore, the second hypothesis is favored to explain the discrepancy between the mutation status in the cells before and after reprogramming.

If the starting populations of H358 and H460 are heterogeneous, and there exists a ‘mutation-free’ subpopulation, why would PCR or Southern-Blot [16] not detect these genes? We propose that the proportion of ‘mutation-free’ subpopulation is too small to provide sufficient template for PCR amplifications to yield products sufficient for ethidium bromide detection. Indeed, we showed this in the dilution of IMR90 genomic DNA of at least 5000 times will mask detection of *CDKN2B* amplicon. Based on this assay, we estimated that there is at most one ‘mutation-free’ cell for every 5000 H358 or H460 cells. Therefore, to attain iPCs derived from this elusive ‘mutation-free’ subpopulation is highly unlikely if the reprogramming process is stochastic. Therefore, we propose that reprogramming of cancer cells follows the Elite model.

### Early tumor progenies may define competency towards reprogramming

Since the reprogramming process discriminates a heterogeneous cancer cell population, we attempted to delineate the underlying characteristics in cancer cells that determines competency towards reprogramming. Previous studies have pointed out that reprogramming factors activate several senescence and tumor-suppressive mechanisms (i.e. *TP53* and *CDKN2A*) that act as barriers towards reprogramming [25]–[27]. Surprisingly, despite somatic gene deletions of these barriers in majority of H358 and H460 cells, none of the randomly picked colonies were null for these genes. In addition, we observed that tumorigenic potential (determined by SCID mice inoculation) weakly emulates the enrichment by the reprogramming process. On the other hand, our data suggests that the derivatives of these iPCs are putative early progenies of the tumor population.

**Figure 5:**
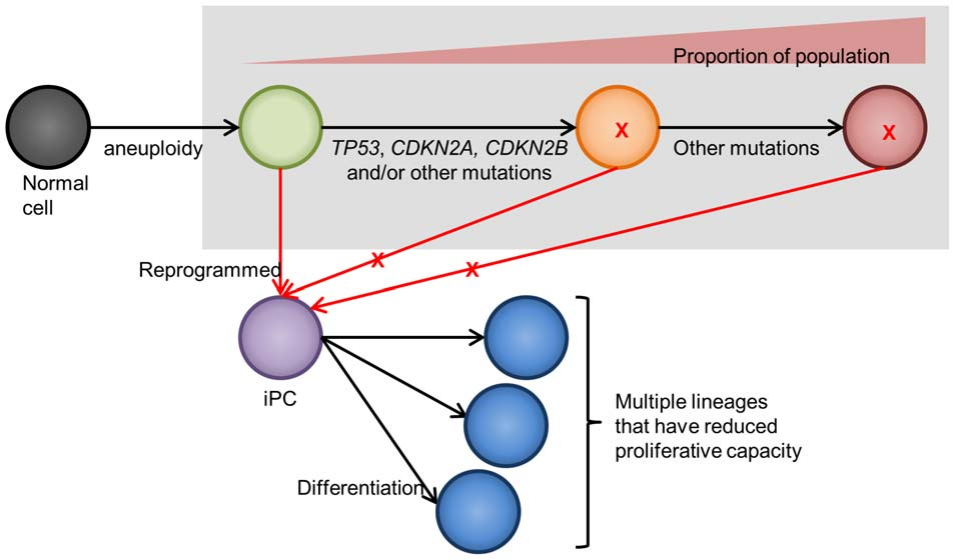
Schematic diagram illustrating the putative origins of the ‘mutation-free’ subpopulation selected for reprogramming. From our data, we observed that the derivatives of our iPCs: 1) lacked key genetic mutation(s); 2) aneuploid; 3) minor subpopulation. Given these observed characteristics, it is safe to assume that these derivatives were early progenies of the tumor population (represented in green). Thus, aneuploidy is possibly first acquired prior to critical mutations to drive further advantageous mutations (demarcated in grey), consistent with the observation by Navin et al (2011). Coupled with the understanding that the pluripotency regulatory circuitry is tightly regulated and complex, less genetic-level insults in cells (circles lacking red ‘X’) will ensure the integrity of the circuitry and thence successful establishment of iPC (represented in purple) that can be differentiated to multiple lineages (represented in blue).

Muller's Ratchet states that mutations in organisms that reproduce asexually are irreversible and accumulates over generations [28]. Similarly, cancer cells ‘reproduce’ via mitosis and genetic damages are irreversible and accumulate over multiple cell cycles. In other words, this implies that the derivatives of iPCH358 and iPCH460 are cells from the earlier stages of tumorigenesis. Moreover, given that the mutated genes in question (*TP53*, *CDKN2A* and *CDKN2B*) are important for the integrity of the genome, it is likely that these ‘mutation-free’ cells have a lower extent of genetic-level insults. Paradoxically, though, these cells are aneuploid and display wider spread of chromosome counts than their parental cells (Figure S2), plausibly corroborating the theory that aneuploidy promotes genomic instability [29]. Concurring with this theory, Navin and colleagues similarly observed that copy number amplification of *KRAS*, an important oncogene, is unique to aneuploid tumor population [14]. Therefore, we propose a guiding hypothesis that reprogramming selects cancer cells from the earlier progenies of tumorigenesis, where genetic-level insults are low but grossly aneuploid (Figure 5). Indeed, genetic integrity maintained by *TP53* and *CDKN2A*, among others, may be necessary in order to preserve the tightly regulated pluripotency circuitry to ensure reprogramming success [30]–[32]. Apart from explaining why iPCs generated from H358 and H460 were not *TP53* null and *CDKN2A* null, respectively, this may answer an intriguing question posed by Zhang and colleagues as to how a reprogrammed sarcoma cell with multiple genetic-level damage could still achieve pluripotency [5]. Future experiments such as Flow-FISH (flow cytometry fluorescence *in situ* hybridization) to sort out the ‘mutation-free’ subpopulation followed by single nucleus sequencing will provide evidence to corroborate or falsify this hypothesis. Additionally, further studies on the genome of explant tumor H358-2 will be of interest in addressing this hypothesis.

### Concluding remarks

In this study, we have utilized cancer cell lines that are inherently heterogeneous, enabling us to observe which variable(s) biases towards the successful generation of iPCs. Indeed, our observations that the ‘mutation-free’ subpopulations were selected against the majority suggest that reprogramming of cancer cells follows the Elite model. This conclusion does not falsify the previous proposal that the generation of iPS follows the Stochastic model, by virtue that normal somatic cells and cancer cells are different. In addition, our data leads to a guiding hypothesis that putative early progenies of the tumor population were selected during the reprogramming process. Future elucidation of associated characteristics to the ‘mutation-free’ subpopulation suggested by our data would be of great utility in further understanding the reprogramming of cancer cells process to unravel the underlying heterogeneous make-up of tumors, which will be key in anti-cancer therapeutic strategies.

## Supporting Information

Figure S1 Expression of CDKN2B protein in H460 and iPCH460. *CDKN2B* is mutated in H460 that renders all PCR assays to fail but did not perturb its protein expression.
(TIF)

Figure S2 Presence of *CDKN2B* in H460 cells from our laboratory and Dr. Koeffler's laboratory (KLab). (A) CDKN2B protein can be detected in H460 cells from our laboratory and KLab. (B) An alternative primer pairs we designed (Table S1) were able to amplify *CDKN2B* in both genomic DNA and cDNA of H460. We sequenced the coding region of this gene and found it to be wild-type (GenBank accession: JX391994).
(TIF)

Figure S3 Metaphase spread shows that post-iPCs (piPCs) are aneuploid. (A) Representative metaphase spreads of H358, H460, piPCH358 and piPCH460. (B) Table summarizing counts of chromosomes from at least eight independent spreads per sample. Blue – DAPI stained chromosomes; green – TRF2; red – CENPA.
(TIF)

Figure S4 2-fold serial dilution of IMR90 genome for amplification of *TP53* and *CDKN2A*. (A) IMR90 genomic DNA was 2-fold serially diluted with H358 genomic DNA as template to amplify *TP53*. We observed that at 4-fold dilution, PCR band corresponding to *TP53* is marginally visible. This gives us an estimate that for every four H358 cells, one is ‘mutation-free’. (B) Similarly, IMR90 genomic DNA was 2-fold serially diluted but instead with H460 genomic DNA. Amplification efficiency of *CDKN2A* was likewise jeopardized by non-specific products; at 32-fold dilution, the PCR band was marginally appreciable thus giving an estimate that for every 32 H460 cells, one is ‘mutation-free’. Regardless, parsing any of these parameters into the probability model in Figure 3 will result in a probability a lot lesser than 0.05.
(TIF)

Presentation S1 Original images used in Figure 1 and Figure 2.
(PDF)

Table S1 Primer Sequences.
(XLSX)

Table S2 Explant Tumor Data.
(XLSX)
