# Supplementary figures and images for: Elite Model for the Generation of Induced Pluripotent Cancer Cells (iPCs)
Source: PLoS One. 2013 Feb 13;8(2):e56702. doi: 10.1371/journal.pone.0056702 (PMC3572060; doi:10.1371/journal.pone.0056702)

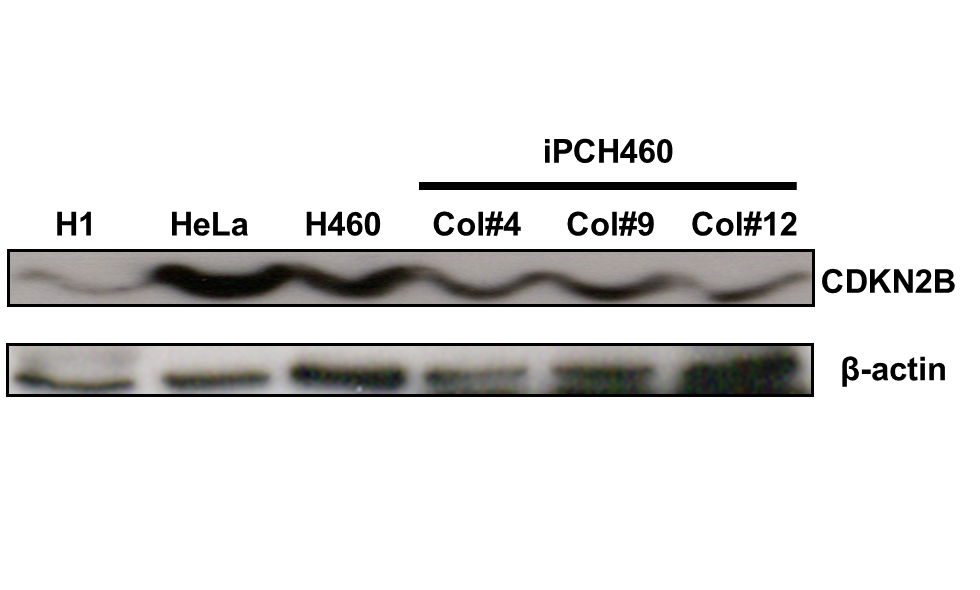

Supplement: Figure S1 — Expression of CDKN2B protein in H460 and iPCH460. CDKN2B is mutated in H460 that renders all PCR assays to fail but did not perturb its protein expression. (TIF) [file pone.0056702.s001.tif]

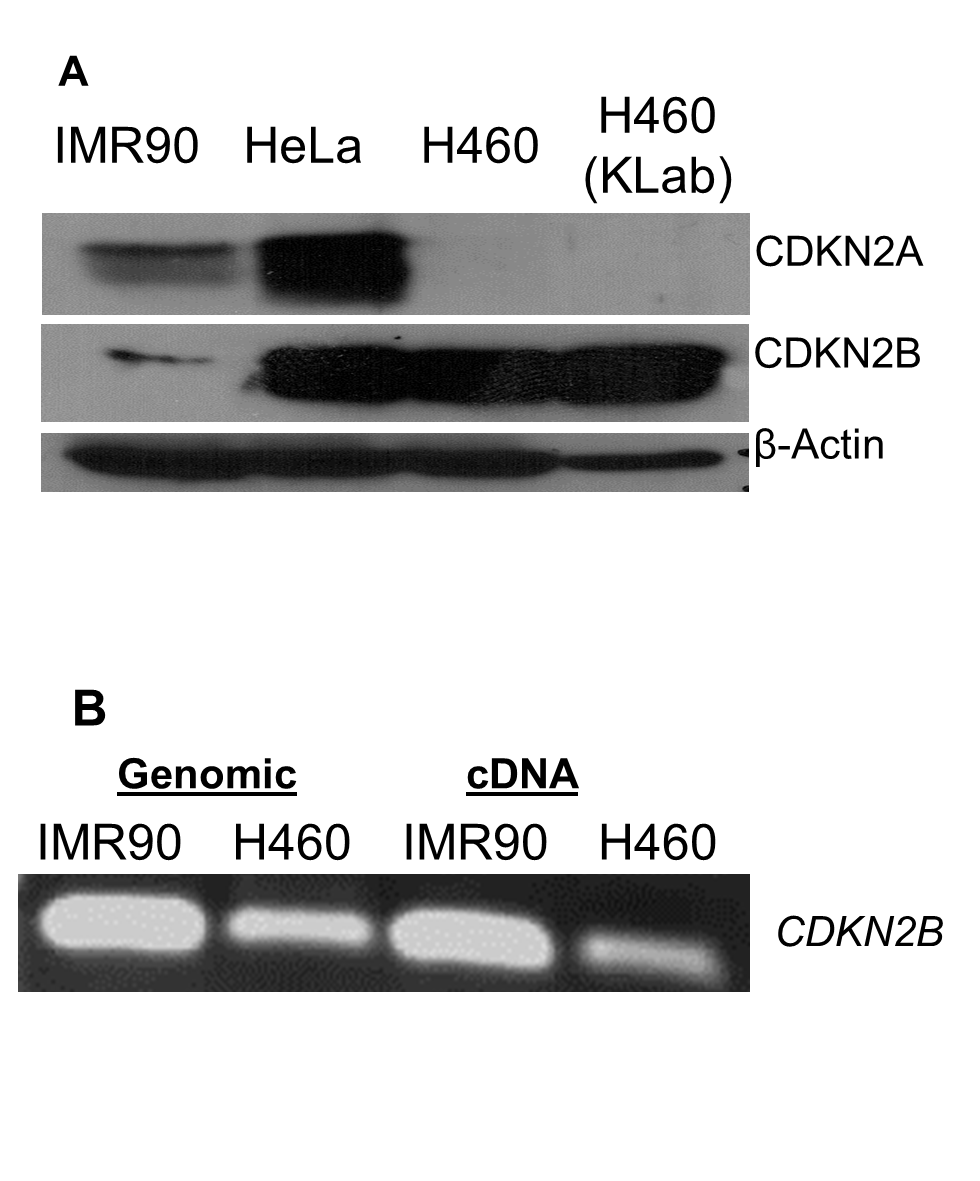

Supplement: Figure S2 — Presence of CDKN2B in H460 cells from our laboratory and Dr. Koeffler's laboratory (KLab). (A) CDKN2B protein can be detected in H460 cells from our laboratory and KLab. (B) An alternative primer pairs we designed (Table S1) were able to amplify CDKN2B in both genomic DNA and cDNA of H460. We sequenced the coding region of this gene and found it to be wild-type (GenBank accession: JX391994). (TIF) [file pone.0056702.s002.tif]

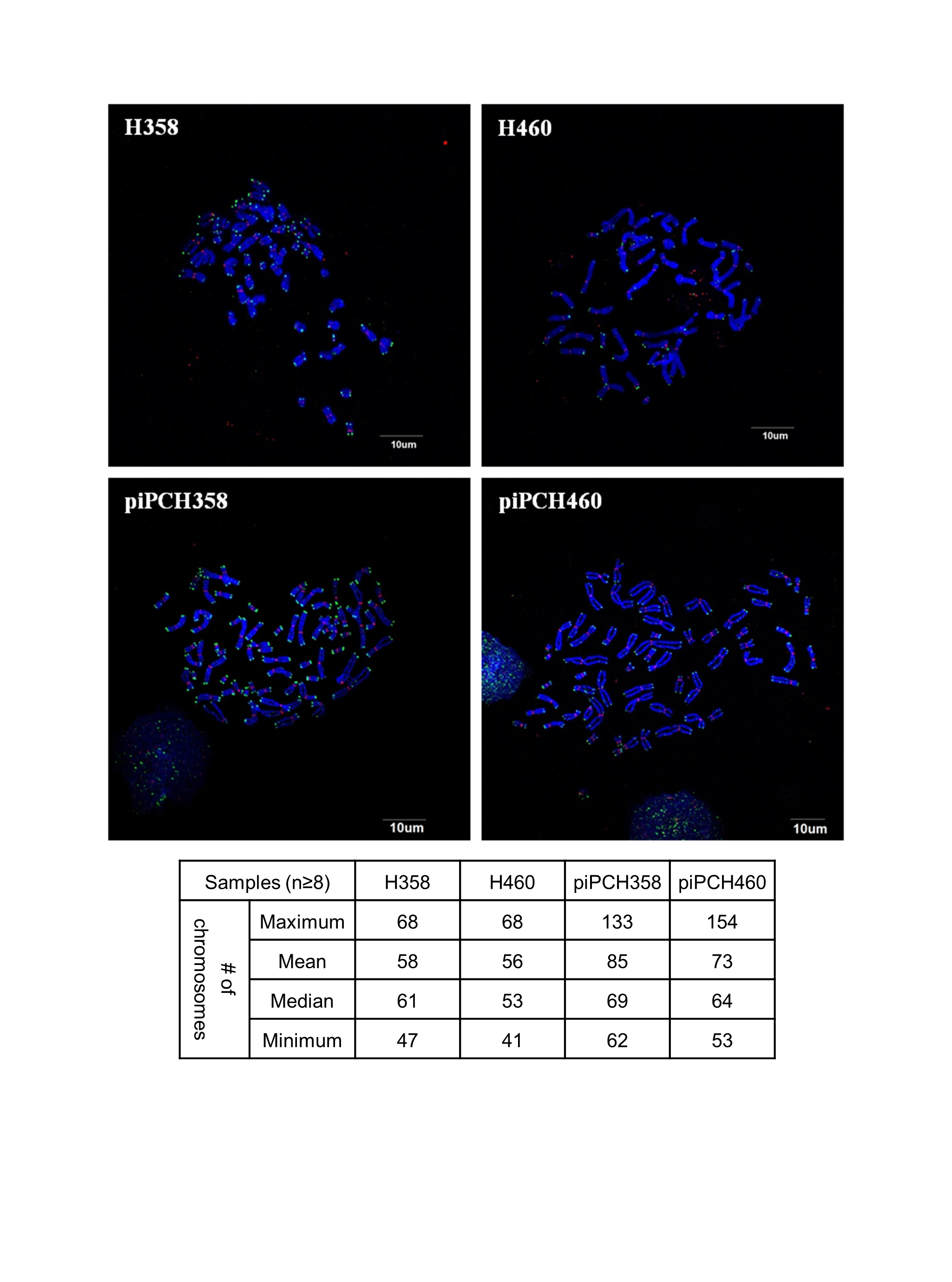

Supplement: Figure S3 — Metaphase spread shows that post-iPCs (piPCs) are aneuploid. (A) Representative metaphase spreads of H358, H460, piPCH358 and piPCH460. (B) Table summarizing counts of chromosomes from at least eight independent spreads per sample. Blue – DAPI stained chromosomes; green – TRF2; red – CENPA. (TIF) [file pone.0056702.s003.tif]

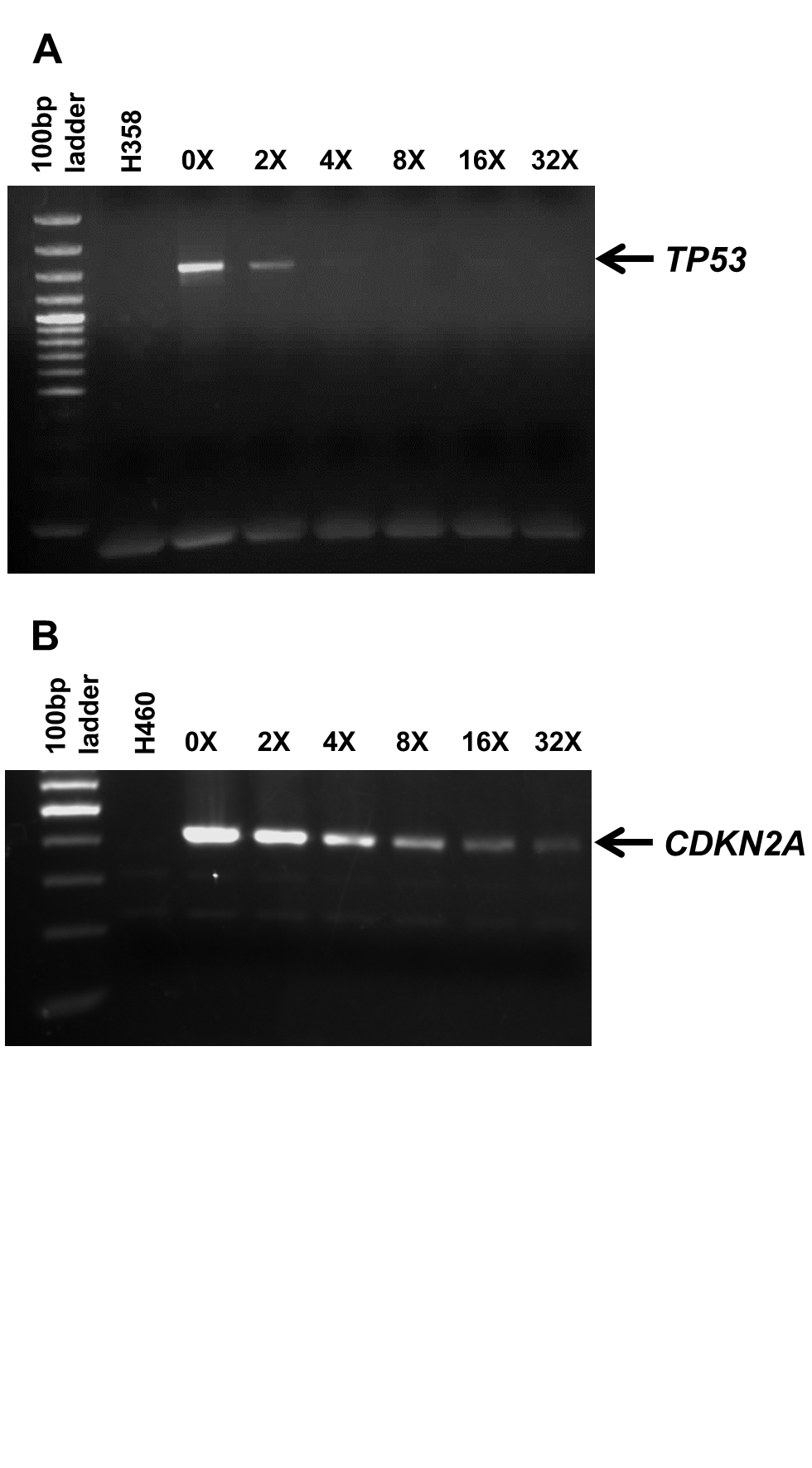

Supplement: Figure S4 — 2-fold serial dilution of IMR90 genome for amplification of TP53 and CDKN2A. (A) IMR90 genomic DNA was 2-fold serially diluted with H358 genomic DNA as template to amplify TP53. We observed that at 4-fold dilution, PCR band corresponding to TP53 is marginally visible. This gives us an estimate that for every four H358 cells, one is ‘mutation-free’. (B) Similarly, IMR90 genomic DNA was 2-fold serially diluted but instead with H460 genomic DNA. Amplification efficiency of CDKN2A was likewise jeopardized by non-specific products; at 32-fold dilution, the PCR band was marginally appreciable thus giving an estimate that for every 32 H460 cells, one is ‘mutation-free’. Regardless, parsing any of these parameters into the probability model in Figure 3 will result in a probability a lot lesser than 0.05. (TIF) [file pone.0056702.s004.tif]
