# Supplementary material for: Elite Model for the Generation of Induced Pluripotent Cancer Cells (iPCs)
Source: PLoS One. 2013 Feb 13;8(2):e56702. doi: 10.1371/journal.pone.0056702 (PMC3572060; doi:10.1371/journal.pone.0056702)
Supplement: Presentation S1 — Original images used in Figure 1 and Figure 2. (PDF) [file pone.0056702.s005.pdf]

TP53

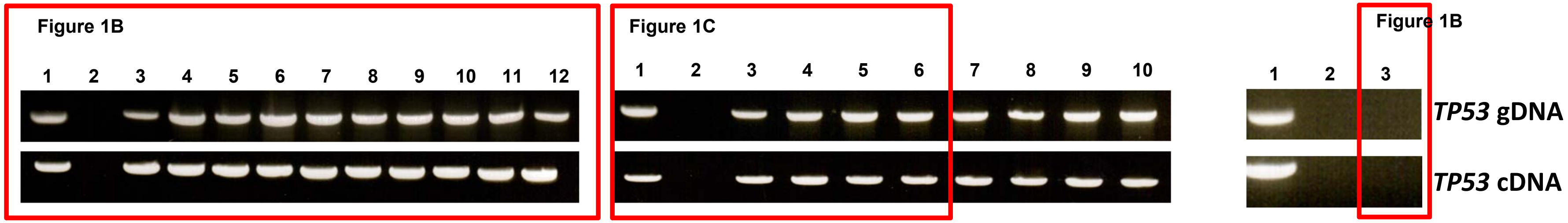

No.	gDNA	cDNA
1	IMR90 P5	IMR90 P5
2	H358 P12	H358 P12
3	H358iPC Col#1 P3	H358iPC Col#1 P3
4	H358iPC Col#3 P3	H358iPC Col#3 P3
5	H358iPC Col#4 P6	H358iPC Col#4 P6
6	H358iPC Col#5 P3	H358iPC Col#5 P3
7	H358iPC Col#8 P5	H358iPC Col#8 P5
8	H358iPC Col#9 P7	H358iPC Col#9 P5
9	H358iPC Col#10 P4	H358iPC Col#10 P5
10	H358iPC Col#11 P3	H358iPC Col#11 P3
11	H358iPC Col#12 P7	H358iPC Col#12 P7
12	H358iPC Col#13 P4	H358iPC Col#13 P4

No.	gDNA	cDNA
1	IMR90 P5	IMR90 P5
2	H358 P12	H358 P12
3	H358iPC Col#3 P33	H358iPC Col#3 P33
4	H358iPC Col#11 P43	H358iPC Col#11 P43
5	H358iPC Col#9 P21	H358iPC Col#9 P21
6	H358iPC Col#1 P22	H358iPC Col#1 P33
7	H358iPC Col#3 P3	H358iPC Col#3 P3
8	H358iPC Col#11 P3	H358iPC Col#11 P3
9	H358iPC Col#9 P7	H358iPC Col#9 P7
10	H358iPC Col#1 P3	H358iPC Col#1 P3

No.	gDNA/cDNA
1	IMR90 P5
2	H358 P12
3	H358 GFP-Control

Slide A. Original images used in Figure 1. Cropped images used in Figure 1 are demarcated in red and its usage is indicated (i.e., Figure 1B). Passage number of the samples used are detailed here and evidently, it does not play a role in affecting the expression of *TP53*.

TP53

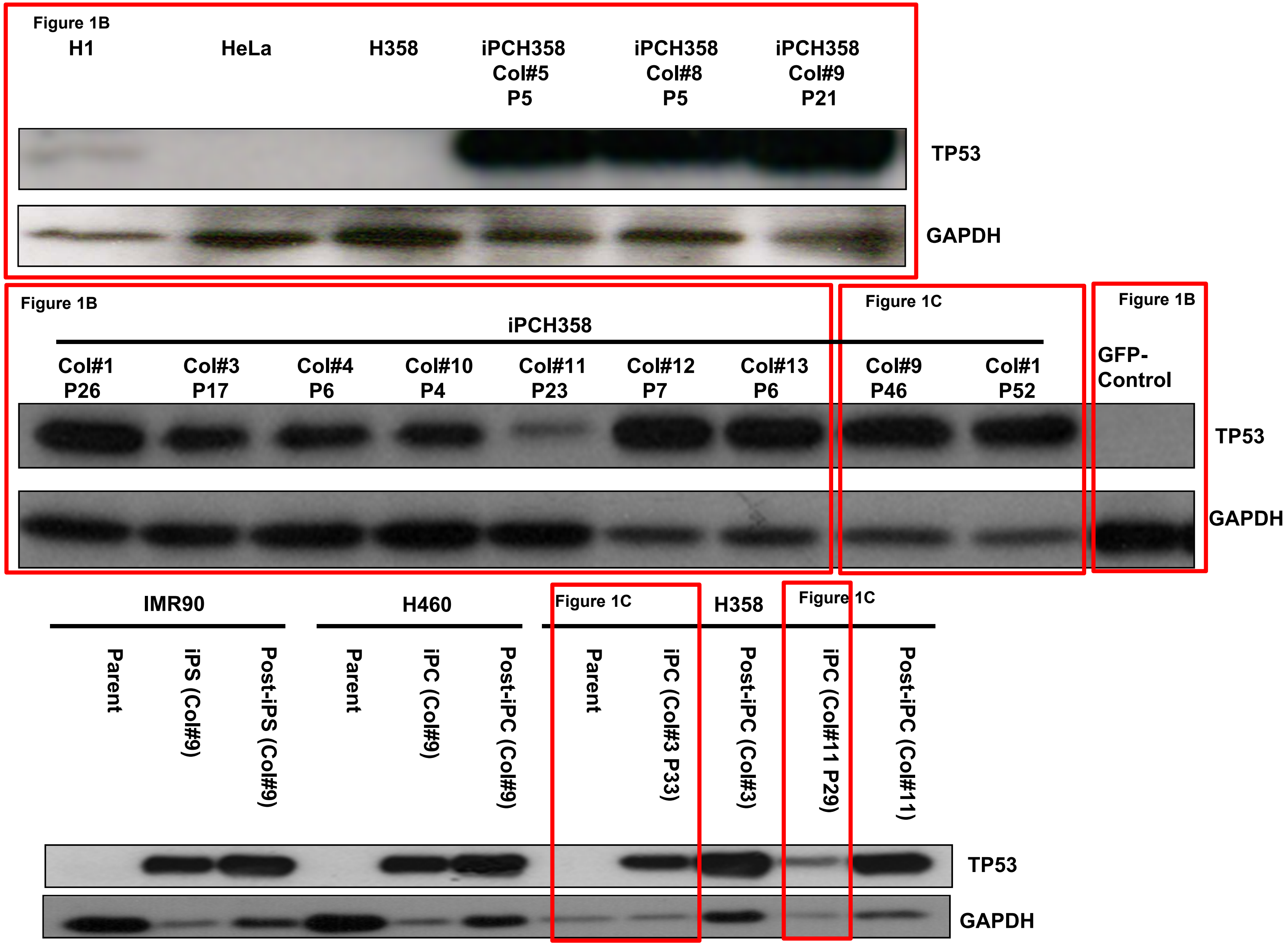

Slide B. Original images used in Figure 1. Cropped images used in Figure 1 are demarcated in red and its usage is indicated (i.e., Figure 1B). Passage number of the samples used are detailed here and evidently, it does not play a role in affecting the expression of TP53.

CDKN2A & CDKN2B

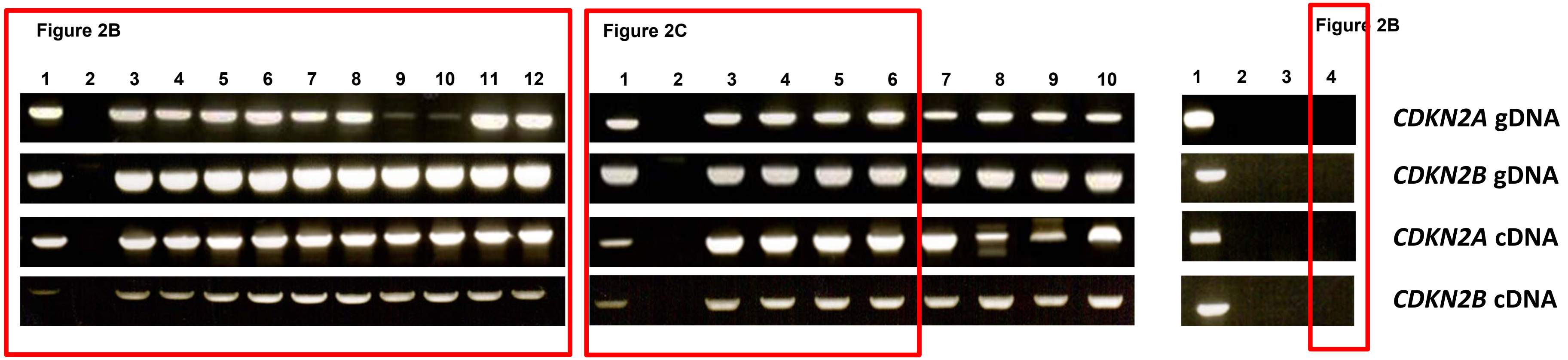

No.	gDNA	cDNA
1	**IMR90 P5**	IMR90 P5
2	**H460 P15**	H460 P15
3	**H460iPC Col#3 P6**	H460iPC Col#3 P6
4	**H460iPC Col#4 P9**	H460iPC Col#4 P9
5	**H460iPC Col#5 P5**	H460iPC Col#5 P5
6	**H460iPC Col#8 P9**	H460iPC Col#8 P9
7	**H460iPC Col#9 P3**	H460iPC Col#9 P3
8	**H460iPC Col#12 P3**	H460iPC Col#12 P3
9	**H460iPC Col#13 P4**	H460iPC Col#13 P4
10	**H460iPC Col#14 P5**	H460iPC Col#14 P5
11	**H460iPC Col#15 P7**	H460iPC Col#15 P7
12	**H460iPC Col#16 P4**	H460iPC Col#16 P4

No.	gDNA	cDNA
1	**IMR90 P5**	IMR90 P5
2	**H460 P15**	H460 P15
3	**H460iPC Col#3 P14**	H460iPC Col#3 P14
4	**H460iPC Col#5 P20**	H460iPC Col#5 P35
5	**H460iPC Col#9 P28**	H460iPC Col#9 P28
6	**H460iPC Col#12 P27**	H460iPC Col#12 P27
7	**H460iPC Col#3 P6**	H460iPC Col#3 P6
8	**H460iPC Col#5 P5**	H460iPC Col#5 P5
9	**H460iPC Col#9 P3**	H460iPC Col#9 P3
10	**H460iPC Col#12 P3**	H460iPC Col#12 P3

No.	gDNA/cDNA
1	**IMR90 P5**
2	**H460 P15**
3	**H460 GFP-control 1**
4	**H460 GFP-control 2**

Slide C. Original images used in Figure 2. Cropped images used in Figure 2 are demarcated in red and its usage is indicated (i.e., Figure 2B). Passage number of the samples used are detailed here and evidently, it does not play a role in affecting the expression of *CDKN2A* and *CDKN2B*.

CDKN2A

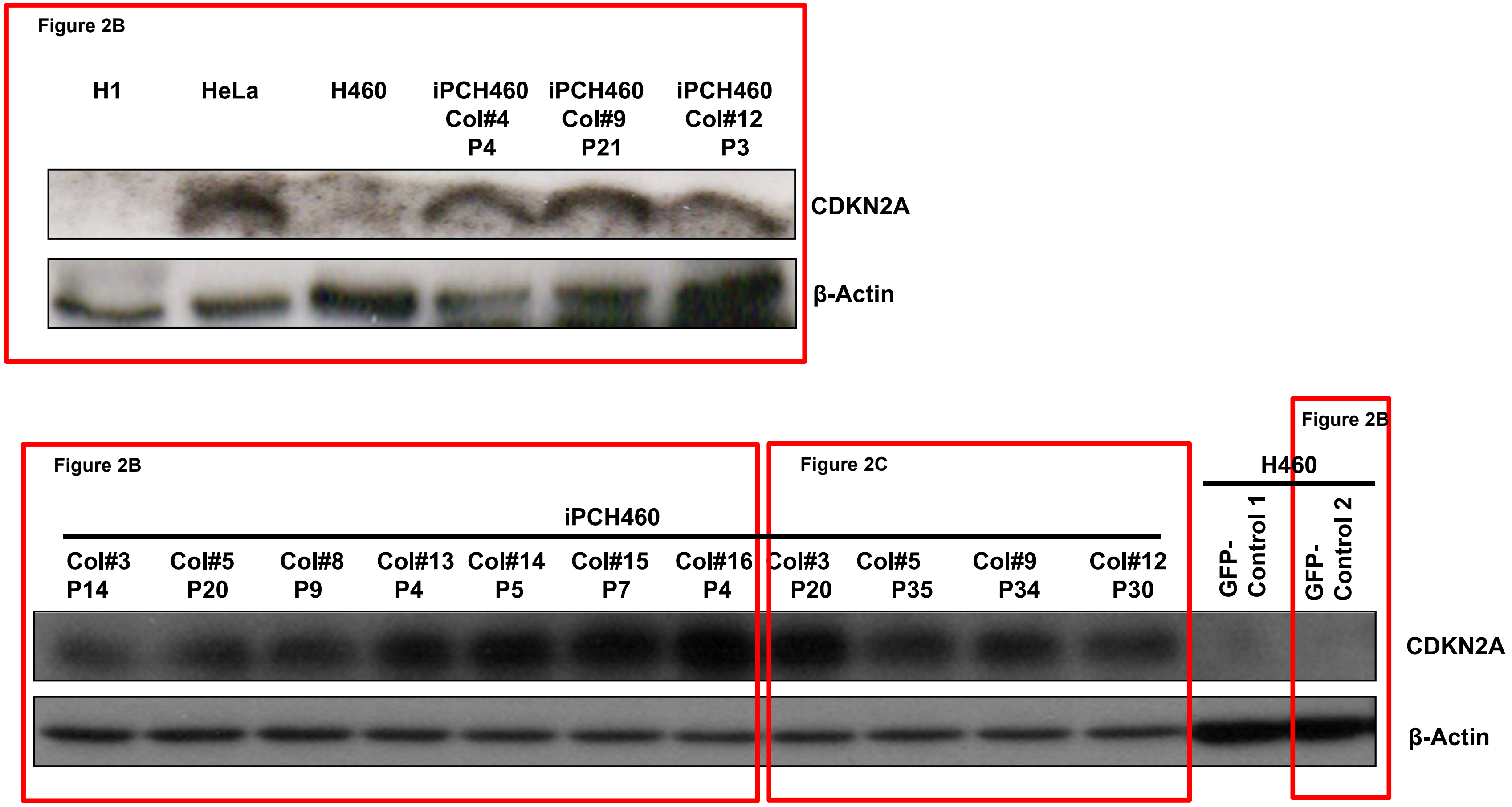

Slide D. Original images used in Figure 2. Cropped images used in Figure 2 are demarcated in red and its usage is indicated (i.e., Figure 2B). Passage number of the samples used are detailed here and evidently, it does not play a role in affecting the expression of CDKN2A.
